# Magnitude of turnover intention and associated factors among nurses working in emergency departments of governmental hospitals in Addis Ababa, Ethiopia: a cross-sectional institutional based study

**DOI:** 10.1186/s12912-020-00490-2

**Published:** 2020-10-14

**Authors:** Andualem Wubetie, Biniyam Taye, Biruk Girma

**Affiliations:** 1 Department of Emergency Medicine, School of Medicine, College of Health Sciences, Addis Ababa University, Addis Ababa, Ethiopia; 2 emergency and critical care nurse in Addis Ababa University TikurAnbesa Hospital, Addis Ababa, Ethiopia

**Keywords:** Addis Ababa, Emergency department, Emergency nurse, Turnover intention

## Abstract

**Background:**

Turnover intention is a probability of an employee to leave the current institution within a certain period due to various factors. It is the strongest predictor of actual turnover expected to increase as the intention increases. Emergency Department (ED) nurses are especially vulnerable to high turnover because of their increased risk of developing burnout and compassion fatigue associated with the work environment. This study is aimed to assess nurses’ intention to leave emergency departments and associated factors at selected governmental hospitals in Addis Ababa, Ethiopia.

**Methods:**

Institutional based cross-sectional study was conducted on 102 nurses in three selected governmental hospitals, Addis Ababa from February 19 to March 31, 2018, using a structured pre-tested self-administered questionnaire. The logistic regression model was used and an adjusted odds ratio with a 95% confidence interval was calculated to identify associated factors.

**Result:**

A total of 102 respondents were involved with a response rate of 91.1%. Among them, 79 (77.5%) respondents had the intention to leave the current working unit of the emergency department or hospital. Significant predictive factors of nurses’ intention to leave their institutions are educational status (adjusted odds ratio (OR) =4.700, 95% confidence interval (CI) = 1.033–50.772; *p* < 0.048), monthly income of less than 3145 Birr (adjusted OR = 6.05, 95% CI = 1.056–34.641; *p* < 0.043) and professional autonomy (adjusted OR = 0.191, 95% CI = 0.040–0.908; *p* < 0.037).

**Conclusion:**

More than 77% of the respondents have the intention to leave their current working place of the emergency unit. Educational status, monthly income, and autonomy were significantly associated with emergency nurses’ turnover intention in three governmental hospitals. Emergency leaders and hospital managers should have made efforts to enhance nurses’ decision making for patient care activities and shared decision overwork or unit related activities.

## Background

Turnover intention is defined as an employee’s own estimated probability of leaving his or her job or current organization at some point in the near future permanently due to various factors. Reasons include dissatisfaction with salary, carrier development opportunity, work environment condition, work overload, and personal factors [1, 2].

In the nursing profession, the turnover of nursing job or professional resigning remains a serious problem associated with the crisis. It resulted in a loss of a trained and qualified employee as transfer, termination, or resignation [3]. Turnover intention is the best actual turnover predictor as expectation increase with intention increases [4]. It is associated with multiple factors including psychological, cognitive, and behavioral components. It is claimed that turnover intention starts as psychological responses to negative aspects of organizations or jobs. The cognitive component involves the decision to leave from the job. Finally, the withdrawal behavior is acted to leave from the current job or actions oriented to future opportunities [5]..

The magnitude of nursing turnover intention is a rapidly-growing global problem affecting the healthcare sector. The studies conducted across the world have shown to be considered a high rate ranging from 18 to 68% [6–10]. It is a major worldwide problem especially in developing countries, including Ethiopia. In previous studies done in different areas of Ethiopia were ranged from50–61% of turnover intention [9, 10].

Turnover of employees is a matter of concern for organizations. It has an impact on the organization including loss of knowledge gained by the employee while on the job, understaffing which in turn leads to decreased effectiveness and productivity of the remaining staff. It has also additional costs related to recruitment and selection, training of new employees, personnel process, and induction [11]..

Emergency nurses are the key components from the emergency team to understand the criticality and breadth of patient care needs to address most efficiently. Thus, emergency nurse turnover has a significant impact on the organization and emergency department leaders who desire to preserve a seasoned and competent nursing workforce [12]. However, they are entering and leaving the emergency department in higher than average numbers, which subjected to cost higher for hospitals to replace staff. The cost for replacement of trained and specialized emergency nurses is higher for advanced training and education of critical competencies needed to provide advanced care for high-acuity patients by the hospital. One in five Registered Nurses within 1 year of hire and one-third within 2 years is estimated to leaves the profession [13].

Studies showed that multiple factors influence the decision of emergency nurses to stay in or leave their workplace. According to the Tourangeau and Cranley model for determinants of nurses’ intention to remain employed, four predictor variables were identified: Job satisfaction, nurses’ characteristics, workgroup cohesion and collaboration, and nurses’ organizational commitment. They reported that control over these four main factors had direct effects on nurse intention to remain employed [14]. Among these; job satisfaction, job stress, work experience, pay and benefit, long shifts, and work-family conflict were identified as variables that could force nurses to leave their workplace [15–18]. According to the Price and Mueller causal nurse turnover model, nurse turnover is directly influenced by nurses’ organizational commitment and indirectly through their job satisfaction, with job satisfaction being influenced by a variety of organizational, demographic, and environmental variables [19].

According to Meyer and Allen’s conceptualization model, organizational commitment is directly linked to an individual’s intention to stay or leave. It has three sub-components; affective commitments, normative commitment, and continuance commitment which was widely used for researchers to study organizational factors [20, 21]. In this model, employees are remained in the organization to achieve the values and goals of the organization is affective commitment. Feeling to have an obligation is normative commitment and lose a lot if they left the organization is continuance commitment. Organizational commitment is an important predictive variable to assess intention to leave the organization [22].

Studies evidenced multiple contributing factors for nurses’ turnover intention. Lower professional title, working in the emergency department, and length of time in the workplace were the factors for turnover intention [23, 24]. A similar study conducted in Indonesia identified factors as workload, schedule and personal reasons were determinants for turnover intention [24].

The ability of hospitals to retain trained and experienced emergency nurses in a cost-prohibitive healthcare environment requires action on behalf of hospital administrators [25].

Emergency Departments (ED) are highly acute patient care environments that are often unpredictable [12]. Nurses working in the Emergency department are highly vulnerable to turnover because of their workload and may have a potential for developing burnout and compassion fatigue [26].

ED nurses with invaluable experience and mentoring capacity may consider leaving the ED for less stressful and physically demanding working conditions. Also, while ED patient volume continues to increase, patient boarding decreases the number of available treatment spaces [27]. This stressful environment is exacerbated by hospitals reducing nursing staff to meet productivity goals, causing some EDs to require mandatory overtime to fill gaps. When nurses must work beyond their shift, at times over 12 h, patient safety is impacted as fatigue makes nurses more prone to errors [28, 29].

Nursing Turnover is continued as a serious concern for healthcare provision and the profession itself. It has an impact on quality of patient care provision, continuity of care interruption, loss of skill full and knowledgeable staff and additional financial costs for replacement and training of staff. It has also a negative impact for staff remained, related with morale and increased workload leading to achieve goal of providing quality care for its patients [30]. Studies have shown considerable potential cost savings where health service managers implement effective strategies to reduce nurse turnover [31].

Understanding more about the interrelationships between individual factors, organizational factors, environmental factors, and job satisfaction with turnover intent can be used by nurse administrators and hospital managers to develop institute based practices designed to increase job satisfaction thus retain nurses [32].

Even if there are limited studies on nurses’ turnover intentions in Ethiopia, they focused on general nurses’ turnover intention. There are no studies conducted in the study area on nurses working in emergency departments that need further studies separately which was initiated to this study.

Thus, there is a great need to conduct an organized study on nurses’ turnover intention on nurses working in ED whose turnover intention is expected higher due to the department’s nature, job stress, over crowdedness and workload. Therefore, this cross-sectional study was aimed to assess the magnitude of turnover intention and associated factors among nurses working in emergency departments of public hospitals in Addis Ababa, Ethiopia. It could provide insights for ED leaders and hospital administrators to develop the appropriate retention strategies and programs for attracting nurses to work in the emergency departments.

## Methods

### Study design and setting

The study was conducted in public hospitals of Addis Ababa city using an institutional-based cross-sectional quantitative study from February 19–March 31, 2018. Addis Ababa (AA) is the capital city of Ethiopia. There are 13 public hospitals in Addis Ababa city. Among thirteen hospitals, three tertiary public hospitals were selected for the study purposively: TikurAnbesa, Menilik II, and St. Paulos Hospitals. These are tertiary hospitals in the city as well as the country where many patients are visited, admitted and treated. They are providing Trauma services not only for Addis Ababa residents but also for the whole country by receiving referral cases from respective regions and city administrations. They also provide general emergency services besides known to be tertiary level hospitals in the city as well as in the country.

### Sample size

A total of 112 nurses were working in the emergency department from the three tertiary level public hospitals. Since the source of population was small (*n* = 112), all nurses from those emergency departments of selected public hospitals were taken as the study subjects.

### Data collection instrument

Turnover intention was the dependent variable which was assessed based on the Tourangeau and Cranley developed model that identifies a set of four predictor variables of nurses’ intention to remain employed: job satisfaction, nurses’ characteristics, workgroup cohesion and collaboration, and nurses’ organizational commitment [13]. Data was collected by using a pretested, structured self-administered questionnaire prepared in the English language which is a medium of instruction for higher education in Ethiopia. The questionnaire was adapted from a previously studied literature [2, 13, 15, 16] and it was modified to for this study (Additional file 1). It was used to assess the organizational factors and job satisfaction which were measured by using five-point Likert scales. The response choices for each item were a 1–5 Likert scale with the choices: 1 = strongly disagree, 5 = strongly agree. Negative items were reverse coded and the answers to show low to a high level for the questions were negatively worded (1-strongly agree, 5 strongly disagree).

The questionnaires were divided into three sub-sections. The first part was used to assess socio-demographic characteristics including sex, marital status, age, monthly income, educational status, dependent family, working experience, and having children living with. The second section was job satisfaction factors that encompass 23 items subscale. It was measured by a 5-point Likert scale in which 5 = very satisfied and 1 = very dissatisfied and the score above the computed mean was taken as satisfied. The reliability of the scale is 0.88. The third section was organizational commitment and its subscale include normative, affective and continuance commitment which was developed and validated by Meyer and Allen (1997). Each subscale was assessed by using three items with a 5-point Likert scale: 1 = strongly disagree and 5 = strongly agree and the scored above-computed mean of the subscale was taken as high commitment. The reliability of the scale is 0.89 for this study. Turnover intention of participants was measured by yes or no item obtained from literature reviews [33–36].

**Pilot study:** The pilot study was conducted to test the clarification of the questionnaire on eleven nurses before the actual data collection in the comparable hospital other than the selected. Some modifications were made to the questionnaires according to the participants’ recommendations. The pilot study results showed that for job satisfaction measurement scale Cronbach’s’ α was 0.88and for organizational commitment was 0.89.

### Data collection process

Before actual data collection, an approval ethical clearance letter was obtained from Addis Ababa university health science college ethical review committee to conduct the study. With the formal ethical clearance letter, we requested each hospital managers, nursing managers, and emergency unit leaders to get permission and all were accepted our request. Then lists of staff nurses working in each emergency department were obtained from head nurses. Nurses who were on vacation, sick leave, clinical nursing experience less than 6 months were exclude from the study. All nurses working in the emergency department of selected hospitals who fulfill the inclusion criteria were included in the study. Four Bachelor of Science (BSc) degree nurses who trained for 2 days on clarification of data tool, collection technique, and quality of data were collected the data. Data collectors informed participants verbally about the study. They distributed the questionnaire to participants for those who agreed to participate. Nurses who were not volunteer were allowed not to participate in the study.

### Data analysis

The collected data were cleaned and entered into the computer using EPI data version 3.1 statistical packages and checked for the consistency of data entry. Data were analyzed using Statistical Package for Social Sciences (SPSS) version 22.0. Cronbach α test was applied to assess the reliability of the data tool. Categorical variables were presented as frequencies and percentages and continuous variables were computed as mean and standard deviation (SD). A bivariate logistic regression analysis was performed to identify the factors that have an association with turnover intention. The statistical significance was at *p*-value < 0.05 with 95% CI.

## Results

### Socio-demographic characteristics of nurses working in the emergency unit

Of the total 112 eligible nurses working in the emergency department, 102 were volunteered to participate in the study making a response rate of 91.1%. Most of them were females (60, 58.8%). The mean age of study participants was 27.4 ± 3.8 ranged from 21 to 45 years. Most of the respondents (84, 82.4%) were a degree holder in nursing, and (77, 75.5%) were single. Seventy-six (74.5%) of them had dependent family they provide financial support. Fifty-one (50%) had less than 2 years of clinical experience in the nursing profession working in the emergency department. The mean monthly income of participants was 4252.26 (±794.921 SD) Ethiopian Birr (ETB) (Table 1).

**Table 1:** Socio-demographic characteristics of nurses working in emergency unit of governmental hospitals of Addis Ababa city, Ethiopia, 2018(*N* = 102)

Variables		Frequency (*N* = 102)	Percent (%)
Sex	Male	42	41.2
	Female	60	58.8
Marital status	Married	25	24.5
	Single	77	75.5
Age	< 25 years	17	16.7
	25–30 years	66	64.7
	> 30 years	19	18.6
Monthly income	< 3145	15	14.7
	3145–3911	18	17.6
	3911–4725	46	45.1
	> 4725	23	22.5
Educational status	Diploma	6	5.9
	Bachelor	84	82.4
	Masters	12	11.8
Had dependent family	Yes	76	74.5
	No	26	25.5
Working experience in ED	< 2	51	50
	2–4	44	43.1
	5–9	7	6.9
Having children living with	Yes	14	13.7
	No	86	84.3

### Turnover intention

Out of the 102 nurses,79 (77.5%) of them had the intention to leave their current health institution of the emergency department.

### Factors related to job satisfaction

The results indicated that most of the participants were unsatisfied with all assessment factors related to job satisfaction. One hundred (98%), 97(95.1%) and 94(92.2%) of the participants were unsatisfied with the income level, workload, and work environment respectively. (Table 2).

**Table 2:** Job satisfaction factors of nurses working in emergency department of governmental hospitals of Addis Ababa city, Ethiopia, 2018(*N* = 102)

Variables	Category	Frequency(n)	Percent (%)
Workplace condition	Unsatisfied	85	83.3
	Satisfied	17	16.7
Workload	Unsatisfied	97	95.1
	Satisfied	5	4.9
Nature of work	Unsatisfied	76	74.5
	Satisfied	26	25.5
Work hour	Unsatisfied	83	81.4
	Satisfied	19	18.6
Organizational commitment	Low	87	85.3
	High	15	14.7
Work environment	Unsatisfied	94	92.2
	Satisfied	8	7.8
Salary level	Unsatisfied	100	98
	Satisfied	2	2
Co-worker relation	Low	61	59.8
	High	41	40.2
Payment per time	Unsatisfied	96	94.1
	Satisfied	6	5.9
Workmate leave hospital	Yes	66	64.7
	No	36	35.3
Workmate leave ED	Yes	74	72.5
	No	28	27.5

### Factors related to organizational commitment

The results showed that about 59.8% and 52.9% of nurses had low affective and normative commitment, respectively, while (53.9%) of them had a high continuance commitment (Table 3).

**Table 3:** Organizational commitment factors associated with turnover intention among nurses in three selected governmental hospitals in Addis Ababa, Ethiopia, 2018 (*n* = 102)

Variable	Category	Frequency	Percent %)
Affective commitment	Low	61	59.8
	High	41	40.2
Normative commitment	Low	54	52.9
	High	48	47.1
Continuance commitment	Low	47	46.1
	High	55	53.9

### Associated factors with nurse’s turnover intention

Bivariable analysis was performed and taking p -value< 0.05 as a cut-off point for determining significance with 95% CI. Six variables showed a significant association with the outcome variable. These were; monthly income, having dependent family members, educational status, Autonomy, professional opportunity, and affective commitment. Socio-demographic characteristics such as age, sex, marital status, and experience did not show a significant association with turnover intention (Table 4).

**Table 4:** Bivariable logistic regression analysis of factors associated with the turnover intention of nurses working in the emergency department of governmental hospitals of Addis Ababa city, Ethiopia, 2018(*N* = 102)

Variables	Category	Turnover intention(*n* = 102)		COR(95%CI	*p*-value
		yes	no		
Sex	Male	18 (42.9)	24 (57.1)	0.857 (0.387,1.897)	0.704
	Female	28 (16.7)	32 (53.3)	1	
Age	< 25 years	9 (52.9)	8 (47.1)	3.150 (0.780,12.727)	0.107
	25–30 years	32 (48.5)	34 (51.5)	2.635 (0.852,8.154)	0.093
	> 30 years	5 (26.3)	14 (73.7)	1	
Marital status	Unmarried	42 (47.7)	46 (52.3)	2.283 (0.665,7.830)	0.189
	Married	4 (28.6)	10 (71.4)	1	
Educational status	Diploma	4 (66.7)	2 (33.3)	**10.00 (1.939,97.500)**	0.048*
	BSC	40 (47.6)	44 (52.4)	4.545 (0.939,22.011)	0.06
	Master	2 (16.7)	10 (83.3)	1	
Work experience	< 2 years	4 (66.6)	2 (33.3)	8 (0.500,127.900)	0.141
	2–4 years	25 (92.6)	2 (7.4)	4.167 (0.434,40.000)	0.216
	5–9 years	16 (38.1)	26 (61.9)	2.462 (0.252,24.020)	0.438
	> 10	1 (20)	4 (80)	1	
Monthly income	< 3145	9 (60)	6 (40)	**4.250 (1.058,17.070)**	0.041*
	3145–3911	8 (44.4)	10 (55.6)	2.267 (0.608,8.447)	0.223
	3912–4727	23 (50)	23 (50)	2.833 (0.947,8.474)	0.062
	> 4727	6 (26.1)	17 (73.9)	1	
Dependent family	Yes	39 (51.3)	37 (48.7)	**2.861 (1.078,7.595)**	0.035*
	No	7 (26.9)	19 (73.1)	1	
Work environment	Unsatisfied	43 (45.7)	51 (54.3)	1.405 (0.317,6.221)	0.654
	Satisfied	3 (37.5)	5 (62.5)	1	
Co-worker relationship	Low	24 (39.3)	37 (60.7)	0.560 (0.252,1.247)	0.156
	High	22 (63.7)	19 (46.3)	1	
Autonomy	Unsatisfied	34 (39.5)	52 (60.5)	**0.218 (0.065,0.732)**	0.014*
	Satisfied	12 (75)	4 (25)	1	
Professional opportunity	Unsatisfied	19 (32.2)	40 (57.8)	**0.281 (0.123,0.642)**	0.003*
	Satisfied	27 (62.8)	16 (37.2)	1	
Scheduling	Unsatisfied	23 (42.6)	31 (57.4)	0.806 (0.369,1763)	0.590
	Satisfied	23 (47.9)	25 (52.1)	1	
Affective commitment	Low	33 (54.1)	28 (45.9)	**2.538 (1.109,5.812)**	0.028*
	High	13 (31.7)	28 (68.2)	1	
Normative commitment	Low	27 (50)	27 (50)	1.526 (0.695,3.353)	0.292
	High	19 (39.6)	29 (60.4)	1	
Countenance commitment	Low	21 (44.7)	26 (55.3)	0.969 (0.443,2.120)	0.938
	High	25 (45.5)	30 (54.5)	1	

* = significant at *P* ≤ 0.05, *COR* Crude odd ratio

### Multivariable binary logistic regression analysis results

Three covariates that were significantly associated (*p* value< 0.05) with turnover intention were fitted into the backward stepwise multivariable binary logistic regression and all three covariates; monthly income, educational status and autonomy were significantly associated factors that appeared in the multivariable model after a stepwise selection process. Diploma nurses were 4.7 times more likely to leave than those who were masters [AOR = 4.700 (95% CI: 1.033–50.772)] and Nurses who had monthly income less than 3145 Ethiopian Birr were 6.05 times more likely to leave than who had a monthly income more than 4725 Ethiopian Birr [AOR = 6.05(95% CI: 1.056–34.641)]. Nurses who were not satisfied with autonomy were 0.2 times more likely to leave than nurses who were satisfied [AOR = 0.191 (95% CI: 0.040,0.908))]. (Table 5).

**Table 5:** Multivariat logistic regression analysis of factors associated with turnover intention of nurses working in emergency department of governmental hospitals of Addis Ababa city, Ethiopia, 2018(*N* = 102)

Variable	Category	Intention to leave(*n* = 102)		AOR(95% CI)	*P*-value
		Yes	No		
Age in years	< 25	9 (52.9)	8 (47.1)	0.343 (0.042,2.815)	0.319
	25–30	32 (48.5)	34 (51.5)	0.759 (0.164,3.501)	0.723
	> 30	5 (26.3)	14 (73.7)	1	
Marital status	Unmarried	42 (47.7)	46 (52.3)	6.364 (0.969,41.778)	0.054
	Married	4 (28.6)	10 (71.4)	1	
Educational status	Diploma	4 (66.7)	2 (73.3)	**4.700 (1.033,50.772)**	0.048*
	BSC	40 (90.9)	4 (9.1)	5.080 (0.585,44.111)	0.141
	MSC	2 (16.7)	10 (83.3)	**1**	
Work experience	< 2	4 (66.7)	2 (33.3)	31.498 (0.504,1968.88)	0.102
	2–4	25 (92.6)	2 (7.4)	5.396 (0.265,109.725)	0.273
	5–9	16 (39.1)	26 (61.9)	3.362 (0.175,64.689)	0.422
	> 10	1 (20)	4 (80)	1	
Monthly income	< 3145	9 (60)	6 (40)	**6.049 (1.056,34.641)**	0.043*
	3145–3911	8 (44.4)	10 (55.6)	1.327 (0.288,6.114)	0.717
	3912–4725	23 (50)	23 (50)	1.749 (0.436,7.018)	0.430
	> 4725	6 (26.1)	17 (73.9)	**1**	
Dependent family	Yes	39 (51.3)	37 (48.7)	2.095 (0.575,7.635)	0.262
	No	7 (26.9)	19 (73.1)	1	
Co-worker relationship	Low	29 (43.9)	37 (56.1)	0.753 (0.260,2.181)	0.602
	High	22 (53.7)	19 (46.3)	1	
Autonomy	Unsatisfied	34 (39.5)	52 (60.5)	**0.191 (0.040,0.908)**	0.037*
	Satisfied	12 (75)	4 (25)	1	
Professional opportunity	Unsatisfied	19 (32.2)	40 (67.8)	0.514 (0.171,1.541)	0.235
	Satisfied	27 (62.8)	16 (37.2)	1	
Affective commitment	Low	33 (54.1)	28 (45.9)	2.322 (0.737,7.313)	0.150
	High	13 (31.7)	28 (68.3)	1	

* = significant at *p* ≤ 0.05, *AOR* Adjusted odd ratio

## Discussions

This study was aimed to assess the magnitude and associated factors of turnover intention among nurses working in the emergency department. It provides an initial step in determining and understanding the work-life of nurses working in emergency department settings. It is an important topic that guides an organization to manage the problem accordingly which might have a negative impact on the ability to meet the patient’s needs and deliver high standards of care. Our study showed that most 79(77.5%) of studied nurses had the intention to leave their current working health institution of emergency department. The finding of this study is higher than the studies done previously. A study conducted in Saudi Arabia on primary health care nurses showed that 40.4% has a turnover intention [15]. Similar studies conducted in different countries like japan 44.3% [37], Republic of Ireland (60%) [6], Lebanon (67.5%) [7], South Africa (51.1%) [38] and Egypt (18.4%) [8] have reported a turnover intention of nurses lower than our study result. It is also higher than the previous studies done Ethiopian region: Sidama zone (50%) [9], east Gojam,(59.4%) [10], and North Shoa Zone Amhara Region (61.3%) [39]. The possible reasons could be that the study site which is done at the emergency department of selected hospitals where there is high patient flow with many critically ill and injured patients are treated. This could result in workload and stress on nurses. It could be the reason that showed most of the nurses are not satisfied with almost all measurement levels of job satisfaction. But it is lower than the study done in Riyadh, Saudi Arabiya (94%) on nurses working in tertiary care hospitals of the same study design. The discrepancy could be the study area of selected hospitals since it was conducted at tertiary care hospitals and advanced care is provided. In this study Concerning the socio-demographic characteristics; educational status and monthly income were associated with nurses’ intention to leave their current working unit of the emergency department. Diploma nurses were more than four times more likely to have the intention to leave than master degree nurses. Advanced training, career development, and continuous learning activities in emergency nursing may promote job satisfaction, increased retention of nurses, and enable the continued provision of high-quality care. This finding is consistent with studies done in Ethiopia in which educational status was significantly associated with turnover intention [40].

Another strong significant predictor of turnover intention in the hospital is monthly income. Nurses whose income less than 3145 Ethiopian Birr monthly were six times more likely they have the intention to leave the emergency unit (AOR = 6.049,95% CI:(1.056,34.641). Socio-demographic characteristic age and work experience were not associated with the nurse’s intention to leave the emergency unit. This finding is in line with a similar study done in Sidama zone public health facilities and also the study was done in southern Ethiopia [9]. No significant difference in turnover intentions by gender was found. In this study, autonomy was significantly associated with nurses’ turnover intention. Nurses who were unsatisfied with autonomy were less likely to leave the hospital than satisfied. This finding is consistent with researches done in that job satisfaction explained as a predictor of intent to leave/stay [41].

Nurses as a professional have the authority to make decisions and the freedom to act per their professional knowledge base. But dissatisfaction of nurses in their autonomy may result in a negative impact in the nursing profession in rapidly changing health care environments. The reason for nurses’ autonomy dissatisfaction may be the working department of highly specialized hospitals where there are strict policies and regulations that strict their practice of autonomy.

Organizational commitment was a significant predictive factor in nurse turnover intention. The assessment finding showed that most nurses working in emergency had low affective and normative commitment which have a negative significant influence on turnover intentions. Low affective commitment means that nurses have a low perception to be part of the hospital organization. They have a low sense of belonging and not happy to be part of the nursing team in these hospitals. It is in line with the theories stated [20–22, 42].

Effective retention mechanisms in healthcare settings can improve employees’ self-esteem and organizational efficiency by advancing the quality of care provision and enhancing the nursing workforce. This high rate of turnover intention indicates that all concerned bodies of the organization should work to develop appropriate and efficient strategies to combat this serious issue and enabling the nurses to perform better care for their patients.

The strengths of this study are that; it can be generalizable to the nurses working in the emergency department of Addis Ababa city governmental hospitals in Addis Ababa city, Ethiopia. In addition, it is the first study done on nurses working at the emergency department which is newly established and given high attention nationwide which can be used as a baseline for the policymakers and further studies. Furthermore, the possibility of non-response bias is very little as the response rate is high (91.1%). The limitations of the study are that it was a cross-sectional and data was collected using self- administer scales. So, further studies can be carried out with more objective instruments. It is better to conduct longitudinal and interventional studies to assess the definite turnover amongst nurses compared with the present study described on turnover intention.

## Conclusions

The overall turnover intention among nurses working in emergency department of public hospitals in Addis Ababa city is high. This is related to multiple factors including monthly income, having dependent family members, educational level, Autonomy, professional opportunity, and affective commitment. Generally, this study indicates that the emergency unit leaders, nursing managers, hospital directors, and hospital affairs should work on the major contributing factors towards the intent to leave emergency nurses’ turnover. In conclusion, effective measures should be taken to improve emergency nurse accomplishment, professional status, and career development to minimize their current turnover intention and to prevent emergency nurses from resigning.

## Supplementary information

**Additional file 1:.** Questionnaires.

## Data Availability

All the data supporting the study findings are within the manuscript. Additional detailed information and raw data are available from the corresponding author on reasonable request.

## Abbreviations

AA: Addis Ababa
BSc: Bachelor of Science
CI: Confidence Interval
ED: Emergency Department
ETB: Ethiopian Birr
RN: Registered Nurse
SPSS: Statistical package for social science
