# Supplementary material for: Magnitude of turnover intention and associated factors among nurses working in emergency departments of governmental hospitals in Addis Ababa, Ethiopia: a cross-sectional institutional based study
Source: BMC Nurs. 2020 Oct 14;19:97. doi: 10.1186/s12912-020-00490-2 (PMC7556577; doi:10.1186/s12912-020-00490-2)
Supplement: Supplementary file 1 — Additional file 1:. Questionnaires. [file 12912_2020_490_MOESM1_ESM.docx]

# Annex III.Questionnaires

ADDIS ABABA UNIVERSITY

COLLEGE OF HEALTH SCIENCES

DEPARTMENT OF EMERGENCY MEDICINE

This is A Questionnaire Designed to collect necessary information for Assessment of Factors Affecting Turnover Intention among Nurses Working in Emergency Departments of Selected Governmental Hospitals in Addis Ababa, Ethiopia.

**Part I. Socio –demographic characteristics**

Sex: 1. Male 2. Female

1. Age in years ------------
2. Marital status

1. Single 2. Married 3.Divorced 4.Widowed

1. Educational statues

1. Diploma 2. BSc 3.Master 4.Other (specify) -------------------

1. How long you have been working in Emergency department? -------------------------------------
2. Monthly income? ---------------------- ET Birr
3. Do you have dependent family? 1. Yes 2, No ————
4. Do you have any children under 18 years living with you? 1. Yes 2. No
5. Did you work more than your contracted hours in your last full working week? (paid or unpaid overtime)?

Yes No

1. Paid overtime [ ] [ ]
2. Unpaid overtime [ ] [ ]
3. If yes, how many extra hours did you work? ----------------.
4. How often do you work more than your contracted hours? (Please tick one box only)
5. Every shift
6. Several times per week
7. Once per week
8. Less than once per week
9. Never
10. Other (please specify) -----------

Part II: Job satisfaction factors

| **Instruction:** There are statements about job satisfaction factors affecting nurses‟ turnover intention and each statement has five alternatives with five point scale. Read each item carefully and circle: **1= very dissatisfied,2=Dissatisfied,3=Neither satisfied nor dissatisfied (neutral),4= Satisfied,5=very satisfied.** | | | | | | | | | | | | | |
| --- | --- | --- | --- | --- | --- | --- | --- | --- | --- | --- | --- | --- | --- |
| No. | | | **Factors** | | Measurement scale | | | | | | | | |
| **Autonomy** | | | | |  |  |  |  |  |  |  |  |  |
| **201** | | | | The extent to make autonomous nursing care decision | 1 | | | 2 | 3 | | | 4 | 5 |
| 202 | | | | The extent to be fully accountable for those decisions | 1 | | | 2 | 3 | | | 4 | 5 |
| 203 | | | | The chance to work alone on the job | 1 | | | 2 | 3 | | | 4 | 5 |
| 204 | | | | The freedom to use your own judgment | 1 | | | 2 | 3 | | | 4 | 5 |
| **Professional opportunities** | | | | | Measurement scale | | | | | | | | |
| **205** | Opportunities for further education/degree or post graduate in nursing | | | | 1 | | 2 | | | 3 | | 4 | 5 |
| **206** | Opportunities to participate in morning rounds | | | | 1 | | 2 | | | 3 | | 4 | 5 |
| **207** | Opportunities to participate in nursing research | | | | 1 | | 2 | | | 3 | | 4 | 5 |
| **208** | Opportunities to write and publish | | | | 1 | | 2 | | | 3 | | 4 | 5 |
| **Scheduling** | | | | | Measurement scale | | | | | | | | |
| **209** | | | | The time available to get through my work | 1 | | 2 | | | 3 | 4 | | 5 |
| **210** | | | | The time available for patient care | 1 | | 2 | | | 3 | 4 | | 5 |
| **211** | | | | Overall staffing levels | 1 | | 2 | | | 3 | 4 | | 5 |
| **212** | | | | The way that I am able to care for patients | 1 | | 2 | | | 3 | 4 | | 5 |
| **213** | | | | The amount of time spent on administration | 1 | | 2 | | | 3 | 4 | | 5 |
| **214** | | | | The amount of time spent talking with my patients | 1 | | 2 | | | 3 | 4 | | 5 |
| **Support** | | | | | Measurement scale | | | | | | | | |
| 215 | | The amount of support and guidance I receive from my supervisor | | | | 1 | 2 | | | 3 | 4 | | 5 |

| Enjoyment | | Measurement scale | | | | |
| --- | --- | --- | --- | --- | --- | --- |
| 216 | I find real enjoyment in my job | **1** | **2** | **3** | 4 | **5** |
| 217 | I consider my job rather unpleasant | **1** | **2** | **3** | **4** | **5** |
| 218 | I enjoy my job more than my leisure time | **1** | **2** | **3** | **4** | 5 |
| 219 | I am often bored with my job | **1** | **2** | **3** | **4** | **5** |
| 220 | I am fairly well satisfied with my job | **1** | **2** | **3** | **4** | **5** |
| 221 | I definitely dislike my job | **1** | **2** | **3** | **4** | 5 |
| 222 | Each day on my job seems like it will never end | **1** | **2** | **3** | **4** | **5** |
| 223 | Most days I am enthusiastic about my job | **1** | **2** | **3** | **4** | **5** |

**Part III:Organizational Commitment**

| **Instruction**: These are statements about nurses’ organizational commitment, and each statement has five alternatives with five point scale. **1= strongly disagree, 2= Disagree, 3= neither agree nor disagree (neutral), 4= Agree, 5= Strongly Agree** | | | | | | |
| --- | --- | --- | --- | --- | --- | --- |
| **No.** | **Nurses’ organizational commitment** | **Measurement scale** | | | | |
| 301 | My organization has a great deal of personal meaning for me | 1 | 2 | 3 | 4 | 5 |
| 302 | I feel a strong sense of belonging to my organization | 1 | 2 | 3 | 4 | 5 |
| 303 | I feel like part of the familyin this organization. | 1 | 2 | 3 | 4 | 5 |
| 304 | I owe a great deal to this organization | 1 | 2 | 3 | 4 | 5 |
| 305 | I would not leave this organization right now because I feel an obligation to stay | 1 | 2 | 3 | 4 | 5 |
| 306 | This organization deserves my loyalty. | 1 | 2 | 3 | 4 | 5 |
| 307 | I feel that I have too few options to consider leaving this organization | 1 | 2 | 3 | 4 | 5 |
| 308 | Too much of my life would be disrupted if I decided to leave this organization now | 1 | 2 | 3 | 4 | 5 |
| 309 | It would be very hard for me to leave this organization right now, even if I wanted to. | 1 | 2 | 3 | 4 | 5 |

##### Part IV: Intention to leave planning

| No | Factors | **Yes** | **No** |
| --- | --- | --- | --- |
| 401 | Do you have a plan to leave Emergency department within the next year? | 1 | 2 |
| 402 | Do you have a plan to stay in emergency departments longer? | 1 | 2 |
| 403 | Do you have been actively asking/demanding/requesting transfer to other departments? | 1 | 2 |
| 404 | Do you have a plan to leave the hospital within the next year? | 1 | 2 |
| 405 | Do you have been actively looking for jobs in other hospital/ organization? | 1 | 2 |
| 406 | Do you have been actively looking for jobs in other profession? | 1 | 2 |
| 407 | Do you have a plan to leave the nursing profession? | 1 | 2 |
| 408 | Do you have training Opportunity? | 1 | 2 |

**THANK YOU FOR YOUR COOPERATION‼!**

FOR DATA COLLECTORS AND SUPERVISORS

1. The results of the questionnaire
   1. Refused
   2. Completed
   3. Incomplete/partially filled
2. Data collector: Name ---------------------------- Code -------------- Sign ---------------
3. Supervisor:Name -------------------------------- Code -------------- Sign ---------------
4. Hospital name -------------------------------- Code ------------------------------
5. Date of data collection ------------------------------------
